# Cooperation in Groups of Different Sizes: The Effects of Punishment and Reputation-Based Partner Choice

**DOI:** 10.3389/fpsyg.2019.02956

**Published:** 2020-01-21

**Authors:** Junhui Wu, Daniel Balliet, Leonard S. Peperkoorn, Angelo Romano, Paul A. M. Van Lange

**Affiliations:** ^1^Beijing Key Laboratory of Applied Experimental Psychology, National Demonstration Center for Experimental Psychology Education (Beijing Normal University), Institute of Developmental Psychology, Beijing Normal University, Beijing, China; ^2^Department of Experimental and Applied Psychology, Vrije Universiteit Amsterdam, Amsterdam, Netherlands; ^3^Max Planck Institute for Research on Collective Goods, Bonn, Germany

**Keywords:** group size, public goods game, reputation-based partner choice, punishment, cooperation

## Abstract

Reputation and punishment are two distinct mechanisms that facilitate cooperation among strangers. However, empirical research on their effectiveness is mainly limited to relatively small groups and does not address how they enhance cooperation in relatively larger groups. We address this gap in the literature by testing hypotheses from competing perspectives about the extent to which reputation-based partner choice and punishment enhance cooperation in both small and large groups. Prior work recognizes that an increase in group size is accompanied by a change in the incentive structure, which determines whether the temptation (extra benefit for each person from non-cooperation over cooperation, regardless of others’ choices) or gain (extra benefit for each person from full cooperation over full non-cooperation) remains constant or varies with group size. Thus, we first test how group size affects cooperation when temptation or gain increases with group size (Study 1), and then move on to testing predictions on the effectiveness of reputation and punishment across different group sizes (Study 2). In Study 1 (*N* = 820), we randomly assigned participants to play an online one-shot public goods game in groups of 4, 20, or 40, while keeping the marginal group return or marginal per capita return fixed across groups, in which case the temptation or gain increased with group size. In Study 2 (*N* = 1,132), we further compared a public goods situation involving a punishment or reputation mechanism with an anonymous situation across group sizes, while the marginal group return was fixed across groups. Overall, we found that when temptation increased with group size, 20-person groups cooperated significantly less than 4-person groups in one-shot interactions, and that this effect was explained by lower expectation of others’ cooperation, less perceived collective efficacy, and greater perceived conflict. However, 40-person and 4-person groups did not vary in one-shot cooperation. Importantly, reputation-based partner choice and punishment invariably promoted one-shot cooperation in groups of different sizes. These findings suggest no simple effect of group size on cooperation and underscore the utility of reputation and punishment in fostering cooperation (at least in one-shot interactions) regardless of the size of groups.

## Introduction

The exponential population growth in human history has facilitated a transition from kin-based small-scale societies to large-scale societies with frequent interactions between strangers (Durand, 1977). This transition may facilitate group living in some aspects (e.g., easier labor division in large ventures), but may also pose challenges to cooperation (e.g., overharvesting). Indeed, cooperation is often costly because it requires individuals to sacrifice their personal interests for the collective good (Rand and Nowak, 2013). However, people often cooperate to benefit their group, and even cooperate with non-kin others on large scales—a puzzle that has attracted extensive attention across disciplines (e.g., Fehr and Gächter, 2002; Tomasello and Vaish, 2013; Perc et al., 2017). To date, a number of mechanisms have been proposed to explain cooperation between strangers, such as reciprocal altruism (Trivers, 1971), indirect reciprocity (Nowak and Sigmund, 2005), costly signaling (Gintis et al., 2001), and social norm enforcement (e.g., costly punishment; Yamagishi, 1986; Clutton-Brock and Parker, 1995; Fehr et al., 2002). In particular, reputation and punishment are assumed to be especially relevant in promoting large-scale cooperation (Panchanathan and Boyd, 2004; Chudek and Henrich, 2011). However, empirical research on these two mechanisms is mainly limited to relatively small groups (e.g., Fehr and Gächter, 2002; Milinski et al., 2002) and does not address how reputation and punishment enhance cooperation in larger groups. In fact, there is scant empirical evidence from behavioral experiments about their effectiveness in groups of different sizes (for one exception in the context of punishment, see Xu et al., 2013).

In this paper, we address this gap in the literature by testing predictions on the effectiveness of reputation and punishment in promoting cooperation in larger (versus smaller) groups. However, studying cooperation in groups of different sizes is complex because an increase in group size is accompanied by changes in the incentive structure (e.g., the individual and group payoff resulting from each member’s behavior). Therefore, we first analyze the incentives to cooperate across groups of different sizes. Next, we forward hypotheses about how the two fundamental pillars of human cooperation—reputation and punishment—may foster cooperation across different group sizes. Finally, we discuss the proximate psychological processes that may account for variations in cooperation across groups.

Theoretically, the larger the group, the more difficult it is to track others’ behavior and to identify free riders, and the more likely that coordination and cooperation would fail due to a lack of group efficacy (Kerr, 1989). Despite a widespread belief that cooperation declines with greater group size (e.g., Dawes, 1980; Van Lange et al., 2013), there is also research showing that larger group size increases (Carpenter, 2007; Szolnoki and Perc, 2011; Barcelo and Capraro, 2015), decreases (Suzuki and Akiyama, 2005), or does not influence cooperation (Kerr, 1989; Zelmer, 2003). This mixed evidence on group size and cooperation may be driven by the payoff structure in a specific interaction (Bonacich et al., 1976; Nosenzo et al., 2015). To illustrate this idea, consider an *n*-person public goods game (*n* ≥ 2). In this game, each person contributes *x* (0 ≤ *x* ≤ *E*) out of the initial *E* monetary units (MUs) to the group account and keeps (*E* – *x*) MUs for themselves. The total contribution is multiplied by *k* (1 < *k* < *n*) and divided equally among *n* persons. The two parameters in this game—marginal group return (MGR = *k*) and marginal per capita return (MPCR = *k*/*n*)—represent the group payoff and individual payoff from each MU contributed. Variations in these parameters determine whether the *temptation* (i.e., the extra benefit for each person from non-cooperation over cooperation, regardless of others’ choices) or *gain* (i.e., the extra benefit for each person from full cooperation over full non-cooperation) remains constant or varies with group size (*n*), and thereby may elicit differences in cooperation (Bonacich et al., 1976). Specifically, when the group payoff (MGR) is fixed, gain remains constant but temptation increases with group size, such that the personal benefit from cooperation decreases in larger groups. In contrast, when the individual payoff (MPCR) is fixed, larger groups produce greater added benefits when all cooperate (vs. no one cooperates), such that temptation remains constant but gain increases with group size, making cooperation more likely to occur in larger groups (see Isaac et al., 1994; Barcelo and Capraro, 2015; Shank et al., 2015). Thus, people may adjust their cooperation level depending on how temptation and gain shift as a function of the size of the interacting group. We predict that people will be *less* cooperative in larger (vs. smaller) groups when temptation increases with group size (*Hypothesis 1a*), but will be *more* cooperative in larger (vs. smaller) groups when gain increases with group size (*Hypothesis 1b*).

As noted earlier, reputation and punishment are two distinct but non-mutually exclusive pathways that facilitate large-scale cooperation among unrelated individuals. Regarding the role of reputation, the theory of indirect reciprocity states that cooperators who gain a good reputation are more likely to be reciprocated by other third parties, and that this process allows cooperation to evolve in large groups of genetically unrelated strangers (Yamagishi and Kiyonari, 2000; Nowak and Sigmund, 2005). Moreover, the theory of competitive altruism posits that people prefer to partner with the best cooperators, who will receive more benefits than less cooperative ones (Barclay and Willer, 2007; Van Vugt et al., 2007). Indeed, prior work reveals that people tend to select and cooperate with partners whose reputation is positive (Capraro et al., 2016), and that reputational cues (e.g., gossip) can effectively promote cooperation (Feinberg et al., 2014; for a review, see Wu et al., 2016b). Thus, we expect more cooperation in a situation with reputation-based partner choice than in an anonymous situation (*Hypothesis 2*).

Is reputation-based partner choice more (or less) effective in promoting cooperation when groups become larger, particularly when temptation increases with group size? Different perspectives have different answers to this question. The theory of competitive altruism suggests that people in larger (vs. smaller) groups would face more competition in selecting the best cooperators as partners and advertising themselves as more cooperative than others (Van Vugt et al., 2007). Similarly, the biological market theory argues that larger groups involve more competition for a good reputation and a lower chance to be chosen as potential partners (Barclay, 2013). Thus, signaling one’s cooperativeness is more important in larger groups than in smaller ones. Notably, costly cooperative behaviors reflect one’s genuine concern for others and thus attract more long-term cooperative partners (Smith and Bliege Bird, 2000). When there is more temptation in larger (vs. smaller) groups (i.e., fixed MGR), cooperation becomes costlier, so reputation-based partner choice should promote cooperation more effectively when implemented in larger (vs. smaller) groups (*Hypothesis 2a*). However, another perspective argues that larger groups are more vulnerable to free riding and involve more difficulty in identifying others’ accurate reputation (e.g., Olson, 1965). Importantly, indirect reciprocity enables cooperation to evolve when the probability of knowing others’ reputation is sufficiently high (Rand and Nowak, 2013), which may be less likely to occur in larger groups. Thus, reputation-based cooperation may be more difficult to evolve as groups become larger (e.g., Suzuki and Akiyama, 2005; dos Santos and Wedekind, 2015). This leads to an opposite prediction that reputation-based partner choice is *less* likely to promote cooperation in larger (vs. smaller) groups when temptation increases with group size (*Hypothesis 2b*).

When individuals live in stable groups where group members have conflicting interests, punishment strategies are likely to evolve to maintain cooperation (Yamagishi, 1986; Clutton-Brock and Parker, 1995). Punishment in this context often involves the intentional imposition of a sanction on a free rider that is costly to the punisher but even more costly to the free rider (typically in a fixed ratio, such as paying one MU to deduct three MUs from the free rider), so it discourages others’ subsequent free riding behaviors. Indeed, people are motivated to punish free riders and norm violators (Ostrom et al., 1992; Fehr and Fischbacher, 2004), and punishment promotes cooperation in both one-shot and repeated interactions (Fehr and Gächter, 2002; for a meta-analysis, see Balliet et al., 2011). Thus, we also expect more cooperation in a situation with punishment than in an anonymous situation (*Hypothesis 3*).

If larger groups cooperate less when temptation increases with group size, as argued earlier, then how does punishment facilitate cooperation in larger groups? The gene-culture coevolutionary theory proposes that naturally occurring intergroup competition creates selection pressures among groups to promote ingroup cooperation and maintain group stability. Such selection pressures would favor altruistic punishment and strong reciprocity (i.e., the tendency to cooperate with others and punish free riders) that are individually costly but benefit the group, and thus allow altruistic punishment and cooperation to sustain in large groups (Bernhard et al., 2006; Henrich and Boyd, 2016; Richerson et al., 2016). In fact, the individual cost of punishing a free rider declines as the number of punishers increases in larger groups (Boyd et al., 2010). Thus, argued from this theory, punishment and social norm enforcement should be more conducive to promoting cooperation in larger (vs. smaller) groups. Indeed, norms and institutions (e.g., punishing free riders) have been shown to facilitate and maintain large-scale cooperation (Boyd et al., 2003; Henrich et al., 2010; Mathew and Boyd, 2011), and people in larger and more complex societies engage in more third-party punishment than those in small-scale societies (Marlowe et al., 2008). However, another perspective on trust and social capital would predict the opposite—punishment more strongly promotes cooperation in smaller groups (Coleman, 1988; Portes, 1998; Ahn and Ostrom, 2008). This perspective suggests that people in high-trust groups believe that others will enforce norms (Coleman, 1988). Moreover, people consider smaller groups as more trustworthy (Wheelan, 2009; La Macchia et al., 2016), and punishment more strongly promotes cooperation in high-trust groups (Balliet and Van Lange, 2013). Thus, we will test whether punishment is *more* effective (*Hypothesis 3a*) or *less* effective (*Hypothesis 3b*) in promoting cooperation in larger groups than in smaller ones when temptation increases with group size.

Group size, as well as reputation and punishment, may affect cooperation through three proximate psychological processes: (a) expected others’ cooperation (Pruitt and Kimmel, 1977), (b) perceived collective efficacy (i.e., group members’ belief that they can solve their problem through collective effort; Kerr, 1989), and (c) perceived conflict of interest (Kelley et al., 2003). First, people in larger groups often feel more uncertain about others’ decisions and thus show lower trust in others (Wheelan, 2009; La Macchia et al., 2016), which predicts less cooperation in social dilemmas (Pletzer et al., 2018). Second, the higher levels of anonymity and uncertainty in larger groups may weaken individuals’ belief that the group can maximize the collective interest through joint effort (Kerr, 1989). Such collective efficacy belief facilitates team performance and group cooperation in highly interdependent tasks (Katz-Navon and Erez, 2005). Third, situations often vary in the degree of corresponding and conflicting interests (Kelley et al., 2003). When temptation increases with group size (i.e., fixed MGR), larger groups involve more conflict of interest, which inhibits cooperation in social interactions (Gerpott et al., 2018). Therefore, when temptation increases with group size, people may expect less cooperation from others, perceive less collective efficacy and more conflict, and thus cooperate less in relatively larger groups, and the opposite may occur when gain increases with group size. Our work will be the first to simultaneously test these psychological processes underlying the effect of group size on cooperation. In addition, we will examine whether the different effects of reputation, as well as punishment, on cooperation across group sizes can be explained by changes in any of these psychological processes.

Taken together, our major goal is to test whether reputation and punishment can promote cooperation more effectively in larger groups where there is a stronger temptation to free ride than in smaller groups. Using a one-shot public goods game, we first examine how cooperation varies with the number of group members (Study 1), and then move on to testing predictions on the roles of reputation and punishment across different group sizes (Study 2). Based on previous work (Isaac et al., 1994; Xu et al., 2013), we used groups of 4, 20, and 40 to represent relatively small, medium, and large groups. Study 1 initially tested the hypothesized group size effect when temptation (i.e., fixed MGR) or gain (i.e., fixed MPCR) increased with group size. Study 2 further compared a public goods situation involving *reputation-based partner choice* or *punishment* opportunities with a *control* condition to test how reputation and punishment promote cooperation across three group sizes. In both studies, we also tested whether differences in expected others’ cooperation, perceived collective efficacy, and perceived conflict could explain the decline in cooperation in larger groups with more temptation (i.e., fixed MGR) and/or the increase in cooperation in larger groups with more gain (i.e., fixed MPCR). Both studies were conducted without deception. All participants provided their informed consent and participated voluntarily.

## Study 1

### Materials and Methods

#### Participants and Design

Based on an *a priori* power analysis (Cohen, 1969, p. 348; Faul et al., 2007), a sample of 540 would result in 80% statistical power to detect a small-to-medium effect (*f* = 0.15) of group size on cooperation. We recruited 820 participants (455 women; *M*_*age*_ = 37.87 years, *SD* = 12.15) in the United States via Amazon Mechanical Turk (MTurk) and randomly assigned them to one of five conditions: 4-person group, 20-person group with either a fixed MGR or a fixed MPCR, and 40-person group with either a fixed MGR or a fixed MPCR^1^. All participants were paid US$1.00, and 34 of them received an extra 2-dollar bonus based on their decisions during the study.

#### Procedure

Participants were randomly assigned into an interacting group of 4, 20, or 40 persons, and were informed to interact with other members online in a decision-making task (i.e., a one-shot public goods game). Each person initially received 20 tokens and decided to contribute any tokens to the group account, while keeping the remaining tokens for themselves. In the 4-person groups, the total contribution was multiplied by 1.6 (i.e., MGR) and then divided equally among four persons. Thus, each person received 0.4 (i.e., MPCR) tokens from each token contributed. In the 20-person and 40-person groups, we kept either the MGR fixed at 1.6 or the MPCR fixed at 0.4. Each token that participants earned in this task represented a 0.05% chance to win a 2-dollar bonus.

To ensure that participants understood the task, they had to correctly answer five comprehension check questions with multiple choices prior to making their decisions. After they made their contribution decisions, they completed the measures of expected others’ cooperation (i.e., “How many tokens on average do you think the other 3/19/39 group members will contribute to the group account?”) and perceived collective efficacy (i.e., “To what extent do you believe that your group can maximize the collective earnings?”; 1 = *not at all*, 7 = *very much*). Then they completed a 30-item Situational Interdependence Scale, including six items that measured conflict (α = 0.84; three items were reverse-coded; e.g., “Our preferred outcomes in this situation are conflicting”; Gerpott et al., 2018) on a 5-point scale (1 = *completely disagree*, 5 = *completely agree*). Their average score across the six items was the measure of perceived conflict. Finally, participants reported their age and gender. We calculated participants’ earnings of tokens based on the payoff parameters and their decisions after randomly composing them into groups of 4, 20, and 40, and then randomly selected 34 bonus winners based on their chance.

### Results and Discussion

#### Group Size Effect on Cooperation

To test our hypotheses about the group size effect, we created four simple contrasts with the four-person group as the reference group: *medium-versus-small contrast* (*fixed MGR or MPCR*), *large-versus-small contrast* (*fixed MGR or MPCR*). A one-way analysis of variance (ANOVA) on cooperation revealed a significant group size effect, *F*(4, 815) = 4.80, *p* = 0.001, η_*p*_^2^ = 0.023. Further planned comparisons revealed a significant medium-versus-small contrast (fixed MGR), *F*(1, 815) = 11.59, *p* = 0.001, η_*p*_^2^ = 0.014, but the other contrasts were not statistically significant (*p*s > 0.23; see Table 1). This indicated that 20-person groups were significantly less cooperative than 4-person groups when temptation increased with group size (i.e., fixed MGR). However, contrary to our predictions, there was no significant difference in cooperation between 40-person and 4-person groups when temptation increased with group size (i.e., fixed MGR), and no significant group size effect on cooperation when gain increased with group size (i.e., fixed MPCR).

**TABLE 1:** Means (and standard deviations) of the key measures in each group size condition (Study 1).

**Measures**	**4-person group**	**20-person group (fixed MGR)**	**20-person group (fixed MPCR)**	**40-person group (fixed MGR)**	**40-person group (fixed MPCR)**
Cooperation	13.63(6.81)^a^	11.02(7.14)^b^	14.08(6.31)^a^	12.71(7.40)^ab^	13.16(6.90)^a^
Expected others’ cooperation	11.48(5.70)^a^	9.64(4.96)^b^	11.00(5.44)^ab^	10.87(5.32)^ab^	11.08(5.47)^ab^
Perceived collective efficacy	5.11(1.64)^a^	4.52(1.69)^b^	5.10(1.61)^a^	4.77(1.75)^ab^	5.02(1.71)^ab^
Perceived conflict	2.25(0.82)^a^	2.49(0.88)^a^	2.27(0.83)^a^	2.38(0.89)^a^	2.25(0.89)^a^
*n*	159	169	162	164	166

*MGR = marginal group return, MPCR = marginal per capita return. In the 4-person groups, MGR = 1.6 and MPCR = 0.4. In the other conditions, either MGR was fixed at 1.6 or MPCR was fixed at 0.4. Means with different superscripts per row for the key measures are statistically different (*p* < 0.05) based on *post hoc* comparisons using Bonferroni corrections.*

#### Expectations, Collective Efficacy, and Conflict

One-way ANOVAs revealed significant group size effects on expected others’ cooperation, *F*(4, 815) = 2.76, *p* = 0.03, η_*p*_^2^ = 0.01, perceived collective efficacy, *F*(4, 815) = 3.75, *p* = 0.005, η_*p*_^2^ = 0.02, and perceived conflict, *F*(4, 815) = 2.48, *p* = 0.04, η_*p*_^2^ = 0.01. Further planned comparisons revealed that only the medium-versus-small contrast (fixed MGR) significantly predicted expected others’ cooperation, *F*(1, 815) = 9.52, *p* = 0.002, η_*p*_^2^ = 0.01, perceived collective efficacy, *F*(1, 815) = 9.94, *p* = 0.002, η_*p*_^2^ = 0.01, and perceived conflict, *F*(1, 815) = 6.18, *p* = 0.013, η_*p*_^2^ = 0.01, but the other contrasts were not statistically significant (*p*s > 0.07). Specifically, when temptation increased with group size (i.e., fixed MGR), participants expected others to be less cooperative, perceived less collective efficacy, and more conflict in 20-person groups (*M*s = 9.64, 4.52, and 2.49) than in 4-person groups (*M*s = 11.48, 5.11, and 2.25), but did not vary in these measures in 40-person and 4-person groups. In addition, when gain increased with group size (i.e., fixed MPCR), there was no significant group size effect on these measures (see Table 1).

#### Mediation Analyses

We further tested whether the three measures mediated the observed group size effect on cooperation using the bootstrapping method based on 5,000 bootstrap samples (Preacher and Hayes, 2008; Hayes and Preacher, 2014). Prior to the analysis, we created four dummy variables (*D*_*mediumMGR*_, *D*_*mediumMPCR*_, *D*_*largeMGR*_, *D*_*largeMPCR*_) with the four-person group as the reference group^2^. The relative indirect effect of *D*_*mediumMGR*_ on cooperation was significant through expected others’ cooperation, *b* = −1.14, 95% confidence interval (CI) [−1.87, −0.43], perceived collective efficacy, *b* = −0.51, 95% CI [−0.90, −0.18], and perceived conflict, *b* = −0.32, 95% CI [−0.64, −0.07]. However, the relative indirect effects of the other three dummy variables on cooperation were not statistically significant through these measures. These results suggested that when temptation increased with group size (i.e., fixed MGR), people in 20-person groups expected less cooperation from others, perceived less collective efficacy and more conflict, and thus cooperated less than those in 4-person groups.

Overall, we only found less cooperation in 20-person (but not 40-person) groups compared with 4-person groups in one-shot public goods game when MGR was fixed. This mixed evidence only partly supported Hypothesis 1a that people cooperate less in larger groups when temptation increases with group size. Hypothesis 1b was not supported, given the null effect of group size when MPCR was fixed. Despite this, our results suggest that having a fixed MPCR across group sizes (i.e., temptation is constant but gain increases with group size) may buffer against a potential negative effect of group size on cooperative behavior. Notably, the observed lower level of one-shot cooperation in 20-person groups than in 4-person groups was explained by lower expectations of others’ cooperation, less perceived collective efficacy, and more perceived conflict.

## Study 2

Study 2 was designed to test how reputation-based partner choice and punishment promote cooperation in groups of different sizes. We also sought to replicate our findings in Study 1 on the group size effect when temptation increased with group size (i.e., fixed MGR) and its underlying psychological processes.

### Materials and Methods

#### Participants and Design

An *a priori* power analysis revealed that a sample of 536 would result in 80% statistical power to detect a small-to-medium interaction effect (*f* = 0.15) between group size and mechanism (three conditions for each; Faul et al., 2007). We used TurkPrime to recruit 1,199 participants in the United States with no experience in our prior study (Litman et al., 2017), and randomly assigned them to one of nine conditions of a three (group size: 4, 20, 40) × 3 (mechanism: reputation, punishment, control) between-participants design. All participants were paid US$1.00, and 16 of them received an extra 2-dollar bonus based on their decisions during the study. Sixty-seven participants attempted to complete the study multiple times before actually completing it and were thus exposed to instructions from different conditions. We excluded these participants from data analyses, leading to a final sample of 1,132 participants (610 women, *M*_*age*_ = 35.05 years, *SD* = 10.91)^3^.

#### Procedure

Participants were randomly assigned into an interacting group of 4, 20, or 40 persons, and were informed to interact with other members online in a one-shot public goods game with two options. Each person decided whether to contribute 20 tokens to a group account or keep these tokens as their own. The total contribution was multiplied by 1.6 and then divided equally among all members. Participants learned that others may make their decisions before or after them, and that decisions within the same group would be matched at the end of the study. The tokens they earned determined their chance to win a 2-dollar bonus.

Afterward, participants were randomly assigned to reputation, punishment, or control condition with different instructions. Participants in the reputation condition learned that their decision would be made public to “some or all members”, and then each member would choose their preferred partner for a new task. They could not continue with this new task if no one selected them as partners (adapted from Barclay, 2004; Van Vugt and Hardy, 2010). Participants in the punishment condition were instructed that after learning about “some or all members’ decisions”, each member could assign up to 10 deduction points to other members. Each deduction point they assigned to others cost them one token but decreased three tokens from others (see also Fehr and Gächter, 2002). Participants in the control condition learned that their decisions were anonymous to others.

Prior to the decision stage, participants had to correctly answer several comprehension check questions with multiple choices within two trials. After they made their contribution decisions, they reported the expected number of cooperators (i.e., “Out of the other 3/19/39 group members, how many of them do you think will contribute 20 tokens to the group account?”), from which we calculated the expected proportion of cooperators to represent expected others’ cooperation. They also completed the measures of perceived collective efficacy (one item) and perceived conflict (six items, α = 0.87) used in Study 1.

**FIGURE 1:**
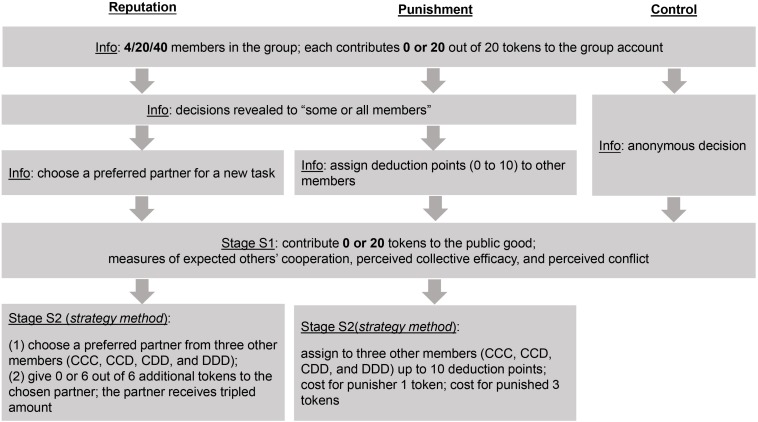
Procedure of Study 2. Participants (a) learned about the payoff structure of the public goods game; (b) read the instructions of the reputation, punishment, or control condition; (c) made their contribution decision and completed relevant measures; and finally (d) made four decisions about partner choice and resource allocation (*reputation* condition) or punishment (*punishment* condition).

Different from the control condition, the reputation and punishment conditions included a second stage during which participants made four decisions assuming that three (out of 3/19/39) other members’ decisions were CCC, CCD, CDD, or DDD (C = contribute, D = do not contribute; *strategy method*)^4^. In this stage, participants in the reputation condition chose their preferred partner to interact with in a new task. In this new task, they were assigned six additional tokens and decided whether to give these tokens to the selected partner. If they did, the partner would receive the tripled amount of 18 tokens (adapted from the indirect reciprocity game in Rockenbach and Milinski, 2006); participants in the punishment condition received an additional 10 deduction points, which they could assign to other members to reduce these others’ earnings (see Figure 1). Finally, participants reported their age and gender. We selected 16 bonus winners based on all participants’ decisions after we randomly composed them into groups of 4, 20, and 40.

### Results and Discussion

#### Cooperation

Overall, 73.14% of all participants cooperated by contributing 20 tokens. After dummy-coding group size into *D*_*medium*_ (20 vs. 4) and *D*_*large*_ (40 vs. 4) and mechanism into *D*_*reputation*_ (reputation vs. control) and *D*_*punish*_ (punishment vs. control), we conducted a hierarchical logistic regression on cooperation (see Table 2). Replicating the results of Study 1, we found a significantly lower cooperation rate in 20-person groups (69.21%) than in 4-person groups (77.46%), *b* = −0.44, Wald χ^2^(1) = 6.90, *p* = 0.009, but no significant difference in cooperation rate between 40-person groups (73.32%) and 4-person groups (77.46%), *b* = −0.24, Wald χ^2^(1) = 1.80, *p* = 0.18. Supporting Hypotheses 2 and 3, both reputation-based partner choice, *b* = 0.82, Wald χ^2^(1) = 23.04, *p*<0.001, and punishment, *b* = 0.53, Wald χ^2^(1) = 11.13, *p* = 0.001, significantly promoted cooperation.

**TABLE 2:** Hierarchical logistic regression on cooperation (Study 2).

	**Step 1**				**Step 2**			
***Predictor***	***b***	***Wald***	***p***	**OR [95% CI]**	***b***	***Wald***	***p***	**OR [95% CI]**
Group size		6.93	0.03			0.47	0.79	
*D*_*medium*_	–0.44	6.90	0.009	0.64 [0.46, 0.89]	–0.13	0.28	0.60	0.88 [0.54, 1.43]
*D*_*large*_	–0.24	1.80	0.18	0.79 [0.56, 1.11]	0.02	0.008	0.93	1.02 [0.62, 1.70]
*Mechanism*		25.54	<0.001			15.94	<0.001	
*D*_*reputation*_	0.82	23.04	<0.001	2.28 [1.63, 3.18]	1.06	10.37	0.001	2.88 [1.51, 5.48]
*D*_*punish*_	0.53	11.13	0.001	1.70 [1.25, 2.32]	1.02	10.86	0.001	2.78 [1.51, 5.11]
Group size × mechanism						4.42	0.35	
*D*_*medium*_ × *D*_*reputation*_					–0.46	1.19	0.28	0.63 [0.27, 1.45]
*D*_*medium*_ × *D*_*punish*_					–0.65	2.59	0.11	0.52 [0.24, 1.15]
*D*_*large*_ × *D*_*reputation*_					–0.17	0.14	0.70	0.84 [0.35, 2.03]
*D*_*large*_ × *D*_*punish*_					–0.73	3.07	0.08	0.48 [0.22, 1.09]

*OR = odds ratio; CI = confidence interval. Cooperation was coded as 0 (contribute 0 tokens) and 1 (contribute 20 tokens). Group size was dummy coded into *D*_*medium*_ (20 vs. 4) and *D*_*large*_ (40 vs. 4). Mechanism was dummy coded into *D*_*reputation*_ (reputation vs. control) and *D*_*punish*_ (punishment vs. control).*

Unexpectedly, we found no significant interactions between group size (*D*_*medium*_ and *D*_*large*_) and mechanism (*D*_*punish*_ and *D*_*reputation*_) in predicting cooperation (*p*s > 0.07; see Table 2). These findings suggest that both reputation-based partner choice and punishment can invariably promote cooperation in 20-person and 40-person groups, in comparison to 4-person groups.

**FIGURE 2:**
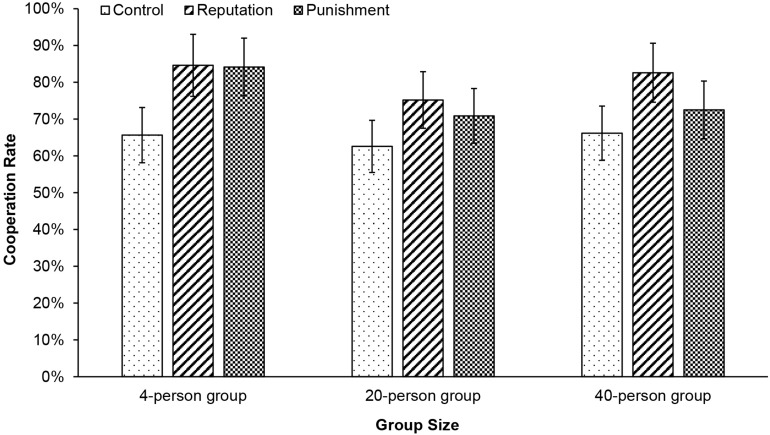
Cooperation rate as a function of group size and mechanism. The cooperation rate was significantly higher in the reputation condition and the punishment condition compared to the control condition, but no significant interaction effect was found. Error bars represent 95% confidence intervals.

Previous evidence suggests that reputation promotes cooperation more than punishment (Grimalda et al., 2016; Wu et al., 2016a). However, other researchers proposed that punishment may be relatively more crucial than reputation and gossip to maintain large-scale cooperation (Jordan et al., 2013). To test both possibilities, we further compared the cooperation rates in the reputation and punishment conditions across groups of different sizes. Prior to the analysis, we re-coded mechanism into *D*_*compare*_ (reputation vs. punishment) and *D*_*control*_ (control vs. punishment) with the punishment condition as the reference group. A similar hierarchical logistic regression on cooperation revealed no significant effect of *D*_*compare*_, *b* = 0.29, Wald χ^2^(1) = 2.56, *p* = 0.11, *D*_*medium*_ × *D*_*compare*_ interaction, *b* = 0.19, Wald χ^2^(1) = 0.16, *p* = 0.69, or *D*_*large*_ × *D*_*compare*_ interaction, *b* = 0.56, Wald χ^2^(1) = 1.29, *p* = 0.26. If anything, the cooperation rate was slightly (but not statistically significantly) higher in response to reputation-based partnerchoice (82.61%) than punishment (72.50%) in 40-person groups, *b* = 0.59, Wald χ^2^(1) = 0.39, *p* = 0.066 (see Figure 2). This pattern of results was consistent when comparing 20-person or 40-person groups with 4-person groups.

#### Expectations, Collective Efficacy, and Conflict

Multivariate analyses of variance revealed that group size had a significant effect on expected others’ cooperation, *F*(2, 1123) = 25.52, *p* < 0.001, η_*p*_^2^ = 0.04, perceived collective efficacy, *F*(2, 1123) = 6.00, *p* = 0.003, η_*p*_^2^ = 0.01, and perceived conflict, *F*(2, 1123) = 6.56, *p* = 0.001, η_*p*_^2^ = 0.01. Mechanism also had a significant effect on expected others’ cooperation, *F*(2, 1123) = 6.53, *p* = 0.002, η_*p*_^2^ = 0.01, perceived collective efficacy, *F*(2, 1123) = 6.56, *p* = 0.001, η_*p*_^2^ = 0.01, and perceived conflict, *F*(2, 1123) = 4.20, *p* = 0.02, η_*p*_^2^ = 0.01. However, there were no significant Group Size × Mechanism interactions on these three measures (*p*s > 0.54). Further planned comparisons revealed that participants in the 20-person and 40-person groups expected others to be less cooperative, perceived less collective efficacy, and perceived more conflict than those in 4-person groups (*p*s < 0.05). These results slightly differed from Study 1 that only revealed significant differences in these measures between 20-person groups and 4-person ones. Moreover, compared to an anonymous situation, both reputation-based partner choice and punishment made participants expect more cooperation from others (*p*s < 0.05), but only reputation-based partner choice could enhance participants’ perceived collective efficacy and reduce conflict (*p*s < 0.01).

#### Mediation Analyses

Similar to Study 1, we further tested whether the observed psychological differences could explain the group size effect on cooperation while coding group size into *D*_*medium*_ and *D*_*large*_. The relative indirect effect of *D*_*medium*_ on cooperation was significant through expected others’ cooperation, *b* = −0.42, 95% CI [−0.63, −0.26], perceived collective efficacy, *b* = −0.19, 95% CI [−0.33, −0.07], and perceived conflict, *b* = −0.20, 95% CI [−0.35, −0.09]. The relative indirect effect of *D*_*large*_ on cooperation was also significant through expected others’ cooperation, *b* = −0.48, 95% CI [−0.69, −0.30], perceived collective efficacy, *b* = −0.13, 95% CI [−0.26, −0.02], and perceived conflict, *b* = −0.12, 95% CI [−0.25, −0.005]. These findings suggest that when temptation increases with group size (i.e., fixed MGR), people expect others to be less cooperative, perceive less collective efficacy and more conflict, and thus cooperate less in relatively larger groups than in smaller ones. In addition, we found that reputation-based partner choice promoted cooperation through expected others’ cooperation, *b* = 0.24, 95% CI [0.10, 0.41], perceived collective efficacy, *b* = 0.20, 95% CI [0.09, 0.34], and perceived conflict, *b* = 0.16, 95% CI [0.05, 0.30], whereas punishment promoted cooperation only through expected others’ cooperation, *b* = 0.15, 95% CI [0.009, 0.31].

Taken together, Study 2 replicated the findings of Study 1 that when temptation increased with group size (i.e., fixed MGR), people cooperated less in 20-person groups than in 4-person groups in one-shot public goods game, and that this was because they expected others to be less cooperative, perceived less collective efficacy and more conflict in 20-person groups than in 4-person groups. Moreover, both reputation-based partner choice and punishment strongly promoted one-shot cooperation regardless of the size of groups.

## General Discussion

Over the past decades, several reviews of social dilemmas have suggested that cooperation tends to decrease with the size of the interacting group (e.g., Dawes, 1980; Van Lange et al., 2013). However, despite some studies that support this conclusion (Suzuki and Akiyama, 2005; Wheelan, 2009), other studies suggest that cooperation increases with group size (Isaac et al., 1994; Carpenter, 2007; Barcelo and Capraro, 2015) or does not relate to group size (Kerr, 1989; Zelmer, 2003). Our research contributed insights into the ongoing debate on the “how and why” of the association between group size and cooperation by taking into account the potential changes in the incentive structure across groups. More importantly, we examined the effective strategies that promote cooperation in groups of different sizes. We addressed these questions across two studies using one-shot public good games. In Study 1, we observed participants’ cooperation in an interacting group of 4, 20, or 40 while keeping the MGR or MPCR fixed across groups, in which case the temptation or gain increased with group size. In Study 2, participants interacting in groups of 4, 20, or 40 with a fixed MGR were further randomly assigned to reputation, punishment, or control condition. This setting allowed us to test hypotheses about the effectiveness of reputation-based partner choice and punishment in relatively small and large groups when the MGR was fixed. Overall, we provide novel evidence that when temptation increases with group size (i.e., fixed MGR), 20-person (but not 40-person) groups cooperate less than 4-person groups in one-shot interactions. Moreover, both reputation-based partner choice and punishment can promote one-shot cooperation in groups of different sizes when temptation increases with group size.

Our first goal was to test whether larger groups would cooperate less when temptation increased with group size (H1a), but cooperate more when gain increased with group size (H1b). Partially supporting Hypothesis 1a, when temptation increased with group size, 20-person groups cooperated significantly less than 4-person groups, yet surprisingly, 40-person and 4-person groups did not vary in cooperation. Unexpectedly, inconsistent with previous research (Barcelo and Capraro, 2015; Shank et al., 2015), we found no statistical difference in cooperation across groups of 4, 20, and 40 when gain increased with group size (i.e., fixed MPCR). Thus, keeping the MPCR fixed may buffer against potential negative consequences of larger groups for cooperation and collective action.

It remains unclear why 40-person and 4-person groups did not vary in cooperation when temptation increased with group size. There are several potential explanations for this unexpected result. First, compared with the 20-person and 4-person groups, the payoff structure in the 40-person group situation may be more cognitively demanding, and thus requires longer decision time and may elicit more dropouts from the study, which may confound with the group size effect. Although we have no data confirming such dropouts, the non-significant difference in the survey completion time across group size conditions (*p*s = 0.93 and 0.08 in Studies 1 and 2) ruled out this alternative explanation. Second, we observed relatively higher levels of cooperation (64.5% of tokens contributed in Study 1 and 73.14% cooperators in Study 2) compared to previous research (e.g., 37.7% of total endowment; for a meta-analysis, see Zelmer, 2003), which may suggest a potential selection bias. Specifically, we speculate that participants are more likely to drop out when interacting in the 40-person groups due to more conflict of interest and lower collective efficacy, and those dropouts are more likely to be low cooperators (e.g., proself individuals who generally prioritize their own interest), while those who completed the survey (especially in the 40-person groups) are more likely to be high cooperators (e.g., prosocial individuals who generally care about the collective good and equality; Van Lange, 1999; Balliet et al., 2009). Since we had no behavioral measures for those dropouts or other relevant measures (e.g., social value orientation, Van Lange, 1999) for all participants, our studies left open this account that future research should address.

Nevertheless, our findings suggest no simple relation between group size, incentives, and cooperation. Although we did not consider all possible group sizes and thus could not determine the optimal size of group that yields the highest level of cooperation, some previous studies can provide insights into this question. For example, Capraro and Barcelo (2015) assigned participants into 12 groups of different sizes (from 3 to 100) and found that 15-person groups were the most cooperative when gain (i.e., benefit for each person when all cooperate than when no one cooperates) increased linearly till groups of 20 and then remained constant. This finding slightly deviates from the argument that larger groups cooperate more when gain increases with group size, which would predict 20-person groups (instead of 15-person groups) to be the most cooperative in this setting. Other field research similarly suggests a non-linear effect of group size on cooperation (e.g., Agrawal and Goyal, 2001; Yang et al., 2013). Importantly, outside experimental settings, many social and ecological factors can drive the optimal size of a group that can manage resources successfully (Casari and Tagliapietra, 2018). Thus, people may not explicitly calculate the costs and benefits of cooperation, especially in complex situations (e.g., larger groups). However, the observed patterns of cooperative behavior across group sizes in our studies were largely consistent with those in the proposed psychological processes underlying cooperation (i.e., expected others’ cooperation, perceived collective efficacy, and perceived conflict). In particular, when temptation increased with group size, people in 20-person groups expected less cooperation from others, had lower belief that their group could maximize the collective interest, and perceived more conflict compared with those in 4-person groups.

Our second goal was to replicate the effect of reputation on cooperation (Feinberg et al., 2014; Wu et al., 2016b) and test hypotheses about how reputation fosters cooperation in relatively small and large groups. One perspective predicts that reputation is more effective in larger groups because people prefer the best cooperators as partners, such that larger groups contain more competition for reputation and less chance to be selected as partners (Van Vugt et al., 2007; Barclay, 2013). Another perspective mainly based on computer simulations posits that larger groups are more vulnerable to free riding, so reputation is less capable of maintaining cooperation (Suzuki and Akiyama, 2007). Our findings suggested that reputation-based partner choice greatly enhanced cooperation and that this positive effect invariably occurred in both relatively small and large groups. Moreover, compared to an anonymous situation, having a reputation mechanism promoted cooperation through eliciting higher expectations of others’ cooperation, greater perceived collective efficacy, and less perceived conflict. Notably, these findings are based on an experimental manipulation of reputation that involves an extra partner choice stage with a resource allocation game. Thus, they do not necessarily contradict with findings from computer simulations that reputation-based cooperation becomes difficult to evolve as group size increases (Suzuki and Akiyama, 2005). Indeed, in simulations that allowed agents to condition their behavior on others’ reputations across many generations, defecting with others whose reputation is bad may make reputation-based cooperation less likely to evolve in larger groups, as one’s defection and bad reputation may elicit others’ defection in turn (Suzuki and Akiyama, 2005). In contrast, participants in our research competed to be chosen by others in the reputation-based partner choice stage. To attract potential partners in this stage, it is important for participants to behave more cooperatively than others, especially in larger groups with more competition over a good reputation.

Our final goal was to replicate the effect of punishment on cooperation (Fehr and Gächter, 2002; Balliet et al., 2011) and test hypotheses on how punishment enhances cooperation in relatively small and large groups. The gene-culture coevolutionary theory suggests that punishment should more strongly promote cooperation in larger groups (Boyd et al., 2003; Henrich et al., 2010), yet it is also plausible that punishment loses its effectiveness in larger groups (Coleman, 1988; Ahn and Ostrom, 2008). We found that punishment invariably promotes cooperation in relatively small to larger groups, which did not support either prediction. Moreover, punishment promoted cooperation only through enhancing one’s belief that others would cooperate. Similarly, previous research shows that public good contribution does not decrease with a larger group size as long as participants can sufficiently monitor and punish many other group members (Carpenter, 2007). Moreover, punishment, at least when it remains intact, induces more trust in others being externally motivated to cooperate (Mulder et al., 2006). Interestingly, we found that the punishment-cooperation relation (odd ratio = 1.70) had a smaller effect size than the reputation-cooperation relation (odds ratio = 2.28). Future research may examine the optimal size of group where reputation can more effectively enhance cooperation and group welfare than punishment.

### Strengths, Limitations, and Directions for Future Research

The present work has some methodological strengths, limitations, and implications for future research. First, due to the inherent difficulties in recruiting people from organizations of different sizes and conducting such a large-scale interaction study in the lab, we chose to recruit relatively large and diverse samples through MTurk and manipulate group size in the experimental setting. Although this might lower our ecological validity, the anonymous online environment guarantees that only group size and payoff parameters (i.e., fixed MGR or MPCR) are the salient cues that participants rely on while making their decisions. Moreover, participants in our studies had a chance to win an extra bonus that was determined by their own and others’ decisions after we randomly composed them into groups at the end of the study. This setup provided them with real incentives to weigh their own interest against others’ interests. One potential limitation of our studies was that participants could not feel others’ physical presence or learn about others’ actual decisions when interacting online. However, some evidence suggests that MTurk participants behave as if their partners are real even when doing so involves a financial cost, and are sensitive to subtle cues about their partners (Summerville and Chartier, 2013), which supports the plausibility of conducting social interaction experiments on MTurk. Moreover, many classic “group effects” (e.g., intergroup contact, bystander effect) have been observed without the physical presence of a group (Garcia et al., 2002; Crisp et al., 2009). Thus, others’ physical presence is not necessary to study group effects on cognition, motivation, and behavior. Nevertheless, future research would enrich our findings by observing real-time interactions in the lab or organizations involving groups of different sizes.

Second, to simplify the procedure in Study 2, we only asked participants to interact once (instead of multiple times) in groups of different sizes, during which they could choose their preferred partner for a new task, punish others after their decisions, or have no other option. In such settings, participants may cooperate due to a motivation to establish a good reputation that increases their chance to be selected by others, or due to fear of punishment. However, when a group of people interact repeatedly, they may also respond differently to others’ behaviors. For example, people may adjust their subsequent cooperation after being (un)selected or punished by others. Moreover, the potential occurrence of antisocial punishment (i.e., punishing people who behave prosocially; Herrmann et al., 2008) and retaliation (i.e., counter-punishing in response to a punishment; Nikiforakis, 2008) in repeated interactions might make cooperation break down. In addition, the use of costly punishment may undermine the collective welfare in repeated interactions (Dreber et al., 2008). Future research needs to test how reputation and punishment may affect cooperation and group efficiency across group sizes in repeated interactions.

Third, different reputation-based strategies (e.g., defecting against or ostracizing free riders) and punishment strategies (e.g., centralized vs. decentralized) may affect cooperation differently. Previous evidence suggests that the possibility of being evaluated may not affect cooperation (Capraro et al., 2016), while reputation-based partner choice (i.e., competitive altruism) can promote cooperation (Sylwester and Roberts, 2013; Giardini et al., 2014), but the strategy of defecting with free riders undermines cooperation (Giardini et al., 2014). Thus, the opportunities to form coalitions with cooperators and to ostracize free riders from one’s current group based on others’ reputation may more robustly promote cooperation than strategies like defecting with free riders. Moreover, punishment can be implemented in a centralized way such that people who contribute less than others, or below a threshold, pay a fine (Kamijo et al., 2014), or in a decentralized way (i.e., group members punish each other). Although centralized punishment is shown to be less effective than decentralized punishment in promoting cooperation (Balliet et al., 2011), the former may be easier to implement and can prevent anti-social punishment and retaliation. Moreover, punishment can be executed by people who cooperate or free ride (Helbing et al., 2010) and can vary in the punishment fine and probability of occurrence (Chen et al., 2015), which may affect the sustainability of cooperation. Notably, different strategies may complement each other in real-life situations. For example, people may coordinate their punishment through gossip with other punishers (Boyd et al., 2010), or switch between punishment and social exclusion in response to free riders (Liu et al., 2018). Thus, it is imperative for future research to test how different reputation-based strategies and punishment strategies enhance cooperation and how they complement each other in groups of different sizes over repeated interactions.

Finally, we only focused on three group sizes with a fixed parameter of MGR or MPCR, while allowing the other to vary. However, different combinations of group sizes and payoff parameters may elicit different perceptions and decisions. Indeed, previous research shows that larger groups cooperate more when MPCR is 0.3, but such effect disappears or is even reversed when MPCR is 0.75 (Isaac et al., 1994; Nosenzo et al., 2015). This pattern of results may be explained by the greater afforded opportunity to exploit others as the individual payoff from each unit of contribution (i.e., MPCR) increases. That is, when people perceive less conflict of interest and believe that others will cooperate, some of them may take this chance to exploit others by withholding their own resources and harvesting the benefit from the group. Future research can manipulate both group size and MPCR to test their potential interaction in predicting cooperation. Moreover, the ways that people are interrelated (i.e., network structure) in groups of different sizes may function differently. For example, people may have small circles of friends for private conversations but larger networks of acquaintances or colleagues for completing large projects (Dunbar, 2004). Variations in network structure and their functions in social groups may affect trust, collective efficacy, and the effective strategies that promote cooperation (Santos et al., 2008; Jiang et al., 2011; Apicella et al., 2012). We believe that future research that combines network structure with reputation and punishment in groups of different sizes would provide useful insights into explaining cooperation within social communities.

## Concluding Remarks

Our research is among the first attempts to test how reputation-based partner choice and punishment foster cooperation in relatively small and large groups. We find that when the temptation to free ride increases with group size, people cooperate less in 20-person (but not 40-person) groups than in 4-person groups in one-shot interactions, which is explained by lower expectations of others’ cooperation, lower perceived collective efficacy, and higher perceived conflict in the interaction. Notably, both reputation-based partner choice and punishment invariably promote one-shot cooperation in groups of 4, 20, and 40 persons, which supports their general effectiveness in promoting cooperation (at least in one-shot interactions). Thus, even in fairly large groups where direct reciprocity has it limits, punishment and reputation mechanisms are prominent solutions that enhance cooperation. We also provide some tentative insights into the psychological mechanisms underlying the effects of reputation and punishment. That is, punishment enhances the diminished trust (i.e., expectation about others’ cooperation) that occurs in larger groups and thus may foster cooperation. It is worth mentioning that reputation-based partner choice promotes cooperation through enhancing both trust and collective efficacy, and reducing perceived conflict across groups of different sizes. These findings provide important insights into how people perceive social interactions involving groups of different sizes and the effective measures that can be taken to promote cooperation in these groups. Taken together, our findings suggest that there is no simple relation between group size and cooperation, but when the temptation to free ride increases with group size in one-shot interactions, reputation-based partner choice and punishment are both effective in promoting cooperation.

## Data Availability Statement

The datasets and syntax for the two studies can be found in the Open Science Framework: https://osf.io/qvys6/.

## Ethics Statement

The studies were reviewed and approved by the Ethics Committee of Faculty of Psychology at Beijing Normal University. Participants provided their informed consent prior to taking part in the studies, and had the opportunity to withdraw at any time during the studies.

## Author Contributions

JW, DB, and PV had the initial idea for the studies and designed the studies. JW collected the data, conducted the data analyses, and wrote the first draft of the manuscript with the generous input by DB, LP, AR, and PV.

## Conflict of Interest

The authors declare that the research was conducted in the absence of any commercial or financial relationships that could be construed as a potential conflict of interest.

## Footnotes

1 One participant who put 1989 as age was treated as age 29 at the time of data collection.

2 *D*_*mediumMGR*_ and *D*_*mediumMPCR*_ compared the 20-person groups with the 4-person groups when MGR was fixed at 1.6 and when MPCR was fixed at 0.4, respectively. *D*_*largeMGR*_ and *D*_*largeMPCR*_ compared the 40-person groups with the 4-person groups when MGR was fixed at 1.6 and when MPCR was fixed at 0.4, respectively.

3 Participants were instructed and aware that they would be screened out of the study if they fail to answer the comprehension check questions correctly after two trials. However, some participants still completed the study through the survey link after they were initially screened out. We paid these participants but excluded them from the data analyses, because they had been exposed to instructions for other conditions in the initial trials. One participant who reported her age as 6 was excluded in the descriptive analysis of age.

4 We used the strategy method (see also Barclay, 2006) with incomplete information about others’ possible decisions to increase our ecological validity, as people in their daily life situations often cannot directly observe all others’ behavior in large groups.
